# Supplementary material for: The Effects of ZnTe:Cu Back Contact on the Performance of CdTe Nanocrystal Solar Cells with Inverted Structure
Source: Nanomaterials (Basel). 2019 Apr 17;9(4):626. doi: 10.3390/nano9040626 (PMC6523567; doi:10.3390/nano9040626)
Supplement: Supplementary file 1 [file nanomaterials-09-00626-s001.pdf]

Electronic Supplementary Information

# The Effects of ZnTe:Cu Back Contact on the Performance of CdTe Nanocrystal Solar Cells with Inverted Structure

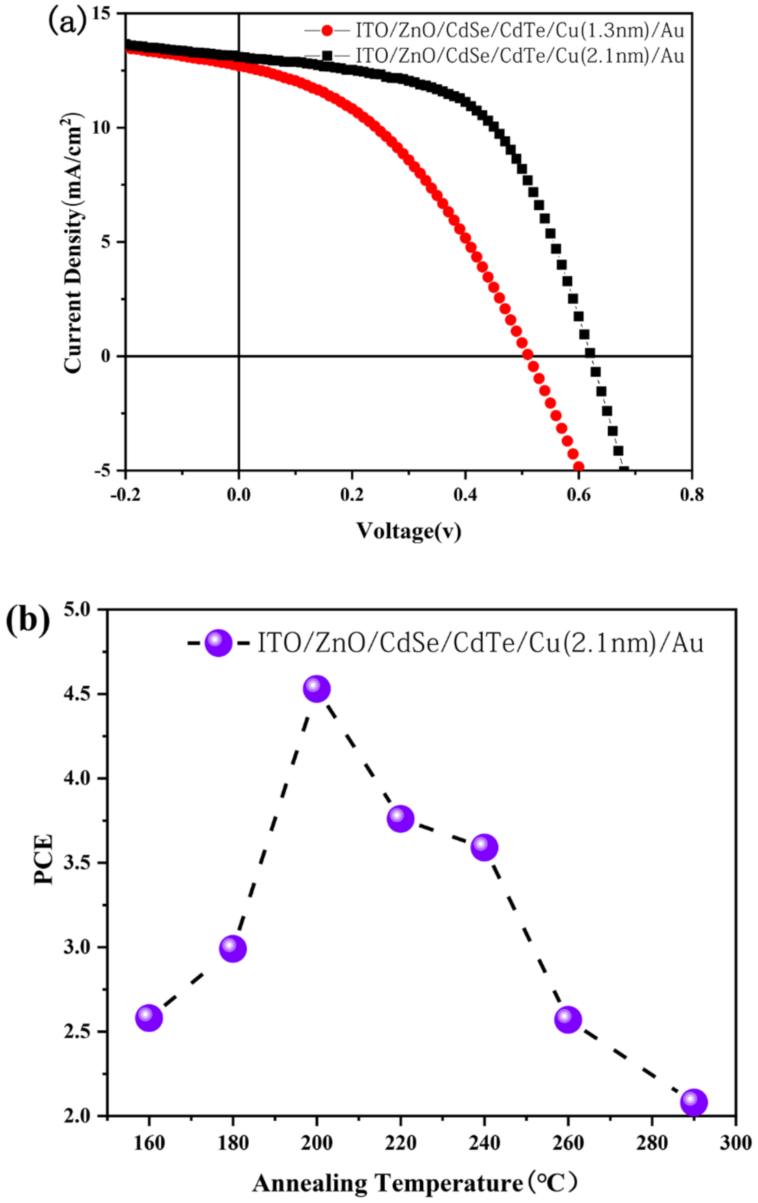


**Figure S1. (a)** *J–V* characteristic of NC solar cells with different thickness of Cu film (all devices annealing at 200 °C); **(b)** *J–V* characteristic of NC solar cells with different annealing temperature (all devices with 2.1 nm Cu film).

**Table S1.** Summary of the photovoltaic parameters of the NC solar cells prepared under different conditions from Figure S1.

| Annealing Temperature (°C) | Cu Layer Thickness (nm) | $V_{oc}$ (V) | $J_{sc}$ $(mA/cm^2)$ | FF (%) | PCE (%) | $R_s$ $(\Omega\cdot cm^2)$ | $R_{sh}$ $(\Omega\cdot cm^2)$ |
|---|---|---|---|---|---|---|---|
| 160 | 2.1 | 0.51 | 12.70 | 39.77 | 2.58 | 19.08 | 204.08 |
| 180 | 2.1 | 0.62 | 9.17 | 52.59 | 2.99 | 18.59 | 442.08 |
| 200 | 2.1 | 0.62 | 13.09 | 55.84 | 4.53 | 12.20 | 324.00 |
| 220 | 2.1 | 0.62 | 11.57 | 52.40 | 3.76 | 16.41 | 323.52 |
| 240 | 2.1 | 0.55 | 14.77 | 44.19 | 3.59 | 16.08 | 276.12 |
| 260 | 2.1 | 0.48 | 10.91 | 49.11 | 2.57 | 20.16 | 284.52 |
| 290 | 2.1 | 0.49 | 9.27 | 45.77 | 2.08 | 17.96 | 237.86 |
| 200 | 1.3 | 0.53 | 8.91 | 49.12 | 2.32 | 19.45 | 196.93 |

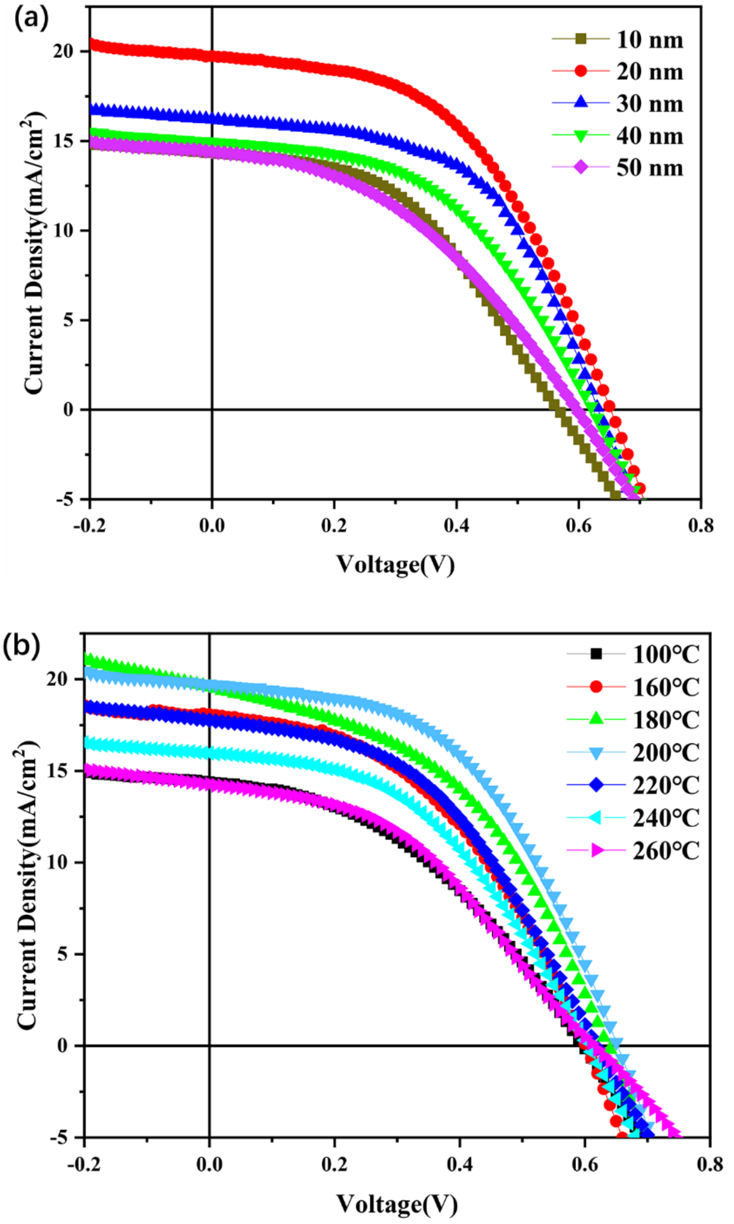


**Figure S2. (a)** *J–V* characteristic of NC solar cells of ITO/ZnO/CdSe/CdTe/ZnTe/Cu (1 nm)/Au structure with different thickness of ZnTe film (all devices annealing at 200 °C); (**b**) *J–V* characteristic of NC solar cells with different annealing temperature (all devices with 20 nm ZnTe film and 1 nm Cu film).
